# Clopidogrel Pharmacogenomics: Validation in a Population of South-Asian Ancestry∗

**DOI:** 10.1016/j.jacadv.2023.100572

**Published:** 2023-08-21

**Authors:** Rolf P. Kreutz

**Affiliations:** Division of Cardiovascular Medicine, Department of Medicine, Indiana University School of Medicine, Indianapolis, Indiana, USA

**Keywords:** clopidogrel, myocardial infarction, pharmacogenomics

**Figure:**
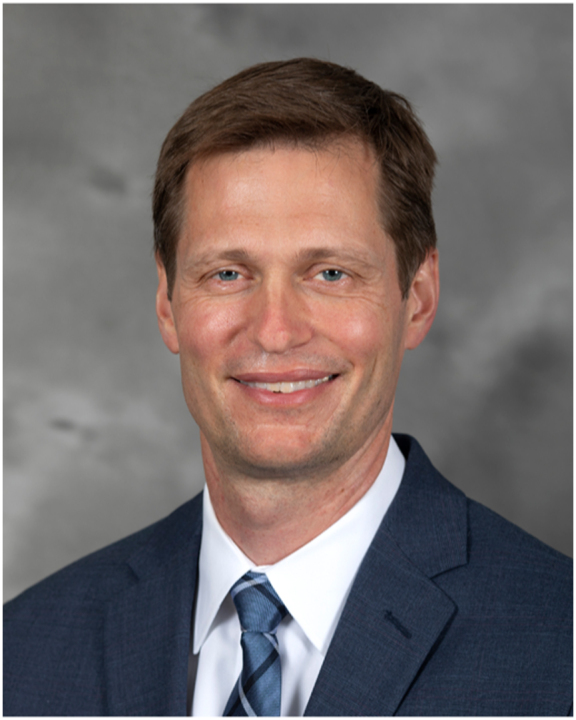

Over the last few years, clopidogrel has become the poster child for personalized medicine in the cardiovascular disease space. Variability in clopidogrel pharmacodynamics among individuals has been consistently linked to adverse clinical thrombotic outcomes in patients with coronary artery disease after percutaneous coronary interventions, although this concept was initially met with hesitancy given the overall benefit of clopidogrel in large double blind randomized trials. The notion that a few individuals with suboptimal therapeutic drug response in a large clinical trial may be acceptable, if the large majority benefit substantially as compared to a blinded control treatment arm has made away in recent decades to ascertain that efficacy is maintained across individuals and populations. As such, pharmacogenomics of clopidogrel is a key example of how trial populations may significantly differ from eventual worldwide use and raise concerns for drug safety and efficacy in populations of varied ancestry. This is true in particular, if variability of therapeutic response due to pharmacogenetic variants is ignored initially during drug development. Among the various enzymes involved in clopidogrel metabolism, cytochrome P450 2C19 (CYP2C19) has been identified as the key enzyme with variability in bioactivity that can significantly contribute to the phenotypic variant of clopidogrel nonresponse, defined as suboptimal generation of active clopidogrel metabolite and consequently insufficient P2Y12 platelet inhibition in carriers of loss-of-function variants of *CYP2C19*. The large randomized double-blind trials that led to approval of clopidogrel for clinical use as well as subsequent trials of novel P2Y12 inhibitors with clopidogrel in the control arm were conducted in largely European ancestry populations (Table 1). The frequency of *CYP2C19* common loss-of-function alleles however varies considerably and is the lowest in European ancestry (∼12%-20%) vs African (∼25%-30%) vs East-Asian ancestry populations (∼40%), and highest reported in select Oceanic populations (62%).^7^ Populations of Indian or Bangladeshi ancestry have been historically neglected for enrollment in large clinical trials; however, South Asians represent increasingly large proportions of the population in western countries such as the United Kingdom or the United States (estimated 5.4 million individuals of South-Asian ancestry living in the United States in 2019).^8^ As such, the findings of the study reported by Magavern et al^9^ in this issue of *JACC: Advances* are noteworthy. They demonstrate that among subjects enrolled in the Genes & Health cohort of Bangladeshi and Pakistani ancestry in the United Kingdom, 57% were carriers of loss-of-function variants, with a very large number of poor metabolizers (13%), which is markedly higher than in previously studied European populations (2.4%). Among poor metabolizers who were treated with clopidogrel the risk of recurrent myocardial infarction was increased more than 3-fold compared to normal metabolizers, and intermediate metabolizers had a 1.5 times higher risk in multivariable logistic regression analysis. This finding is consistent with the HR for stent thrombosis and myocardial infarction observed among poor metabolizers treated with clopidogrel in other studies, including those of East-Asian ancestry.^10^^,^^11^ Given the very large prevalence of poor metabolizers and large number of patients of Bangladeshi and Pakistani origin who receive prescriptions for clopidogrel worldwide, the results of this study are a call to action for clinicians who treat these patients. The Tailored Antiplatelet Initiation to Lessen Outcomes Due to Decreased Clopidogrel Response after Percutaneous Coronary Intervention (TAILOR-PCI) study failed to demonstrate a significant reduction in the primary end point (death, myocardial infarction, stroke, severe recurrent ischemia, stent thrombosis) at 1 year with *CYP2C19* genotyping guidance for clopidogrel therapy (HR: 0.66; 95% CI: 0.43-1.02; *P* = 0.06); however, there was a significant reduction in risk of the primary end point within the first 3 months after randomization in post hoc analysis (HR: 0.21; 95% CI: 0.08-0.54; *P* = 0.001).^5^ If applied to patients of Bangladeshi or Pakistani origin, a 3-fold higher risk reduction could likely be expected given the roughly 3-fold higher prevalence of poor metabolizers. Similarly, the POPular Genetics study showed a nonsignificant reduction in the risk for net clinical combined end point (death, myocardial infarction, definite stent thrombosis, stroke, or major bleeding) with genotype guided P2Y12 therapy vs standard therapy (HR: 0.87; 95% CI: 0.62-1.21; *P* = 0.40), but a significant reduction in bleeding events (HR: 0.78; 95% CI: 0.61-0.98; *P* = 0.04).^6^

**Table 1:** Patient Enrollment in Pivotal Clinical Trials of Clopidogrel According to World Region

Study	North America	Europe	South America	Australia/New Zealand	Africa	East Asia	South Asia
CAPRIE^1^	40%	52%	0%	8%	0%	0%	0%
CURE^2^	20%	62%	9%	7%	2%	0%	0%
TRITON-TIMI 38^3^	32%	51%	4%	14% (Middle Eastern/Pacific/Africa/Asia)			
PLATO^4^	10%	74%	6%	0.4%	1%	6%	3%
TAILOR-PCI^5^	75%	0%	0%	0%	0%	25%	0%
POPular Genetics^6^	0%	100%	0%	0%	0%	0%	0%

Clopidogrel remains the most prescribed P2Y12 inhibitor world-wide due to significantly lower cost than ticagrelor and prasugrel, and therefore the impact of vulnerability to clopidogrel nonresponse is extremely significant particularly in populations with high prevalence of *CYP2C19* loss-of-function variants in the context of secondary prevention after myocardial infarction. Cost-effectiveness analyses of preemptive pharmaco-genotyping for clopidogrel need to include modifications to account for particularly at-risk subpopulations. Future efforts at implementation of personalized medicine treatment approaches will require to include populations of various ancestry that may only subsequently be identified as particularly vulnerable to variability in drug response.

In conclusion, the current manuscript by Magavern et al adds important data to the growing evidence that *CYP2C19* poor or intermediate metabolizers treated with clopidogrel are at increased risk for recurrent myocardial infarction and supports the use of pharmacogenomic testing for clopidogrel resistance in at risk patient populations. It highlights the importance of considering preemptive pharmaco-genotyping in patients of South-Asian origin, particularly those of Bangladeshi and Pakistani ancestry.

## Funding support and author disclosures

Dr Kreutz has received research funding from 10.13039/501100016198Idorsia and 10.13039/100004326Bayer; and has received consulting fees from Haemonetics.
